# Supplementary material for: Carrier-Mediated Process of Putrescine Elimination at the Rat Blood–Retinal Barrier
Source: Int J Mol Sci. 2023 May 19;24(10):9003. doi: 10.3390/ijms24109003 (PMC10219508; doi:10.3390/ijms24109003)
Supplement: Supplementary file 1 [file ijms-24-09003-s001.zip › ijms-2395529-supplementary.pdf]

Supplemental Information

Culture medium

- **TR-iBRB2 cells**

DMEM with 10% fetal bovine serum, 20 mM $NaHCO_3$, 70 mg/L benzylpenicillin, and 100 mg/L streptomycin

- **RPE-J cells**

DMEM with 4% fetal bovine serum, 20 mM $NaHCO_3$, 25 mM D-glucose, 0.1 mM nonessential amino acids, 60 mg/L benzylpenicillin, and 125 mg/L streptomycin

Buffer contents

- **Extracellular fluid (ECF) buffer**

122 mM NaCl, 25 mM $NaHCO_3$, 3 mM KCl, 1.4 mM $CaCl_2$, 1.2 mM $MgSO_4$, 0.4 mM K_2HPO_4, 10 mM D-glucose, and 10 mM HEPES, pH 7.4

- **Na^+-free ECF buffer**

122 mM LiCl, 25 mM $KHCO_3$, 3 mM KCl, 1.4 mM $CaCl_2$, 1.2 mM $MgSO_4$, 0.4 mM K_2HPO_4, 10 mM D-glucose, and 10 mM HEPES, pH 7.4

- **Cl^--free ECF buffer**

122 mM Sodium gluconate, 25 mM $NaHCO_3$, 3 mM potassium gluconate, 1.4 mM calcium gluconate, 1.2 mM $MgSO_4$, 0.4 mM K_2HPO_4, 10 mM D-glucose, and 10 mM HEPES, pH 7.4

- **K^+-replacement ECF buffer**

125 mM KCl, 25 mM $KHCO_3$, 1.4 mM $CaCl_2$, 1.2 mM $MgSO_4$, 0.4 mM K_2HPO_4, 10 mM D-glucose, and 10 mM HEPES, pH 7.4

- **Standard oocyte saline (SOS) solution**

100 mM NaCl, 2 mM KCl, 1.8 mM $CaCl_2$, 1 mM $MgCl_2$, 5 mM HEPES, 25 μg/mL gentamycin, 2.5 mM pyruvate, and 1% bovine serum albumin, pH 7.5

- **ND96 solution**

96 mM NaCl, 2mM KCl, 1mM MgCl, 1.8 mM $CaCl_2$, and 5 mM HEPES, pH 7.4

Statistical analysis

Statistical analyses were carried out using a one way analysis of variance (ANOVA) followed by Dunnett's test and an unpaired two-tailed Student's t-test for several and two groups, respectively. Unless otherwise indicated, all data represent means ± S.D.

Table S1. Primers for full-length cDNA cloning

Genes	Gene Bank Accession #	Orientation	Primer sequence (5' to 3')	Restriction Enzyme
rCTL1	NM_053492.3	Forward	TCTAGAATGGGCTGCTGCAGCTCCGCC	*Xba* I
		Reverse	AAGCTTTCACCTTTTCCTGAGCATCGGCTT	*Hind* III
rCTL3	NM_001013914	Forward	TCTAGATTCATCATGGGTTACTCGGTGGT	*Xba* I
		Reverse	AAGCTTCTATCTCACAATGGCCGGAGTT	*Hind* III
rCTL4	NM_212541	Forward	TCTAGATGAGCCATGGGGAAAAAGCA	*Xba* I
		Reverse	GCAGTCGTGGGTCACGATGA	*Not* I

Table S2. Primers for RT-PCR analysis

Target mRNA	Gene Bank Accession #	Orientation	Primer sequences (5' to 3')	Product size (bp)
rCTL1	NM_053492.3	Forward	TCTAGAATGGGCTGCTGCAGCTCCGCC	162
		Reverse	AAGCTTTCACCTTTTCCTGAGCATCGGCTT	
rCTL2	NM_001134715.1	Forward	ATGTGCTCCATGCTCTACC	176
		Reverse	TTCAATGGGAAGGTCTCTGG	
rCTL3	NM_001013914	Forward	TCTAGATTCATCATGGGTTACTCGGTGGT	243
		Reverse	AAGCTTCTATCTCACAATGGCCGGAGTT	
rCTL4	NM_212541	Forward	TCTAGATGAGCCATGGGGAAAAAGCA	276
		Reverse	GCAGTCGTGGGTCACGATGA	

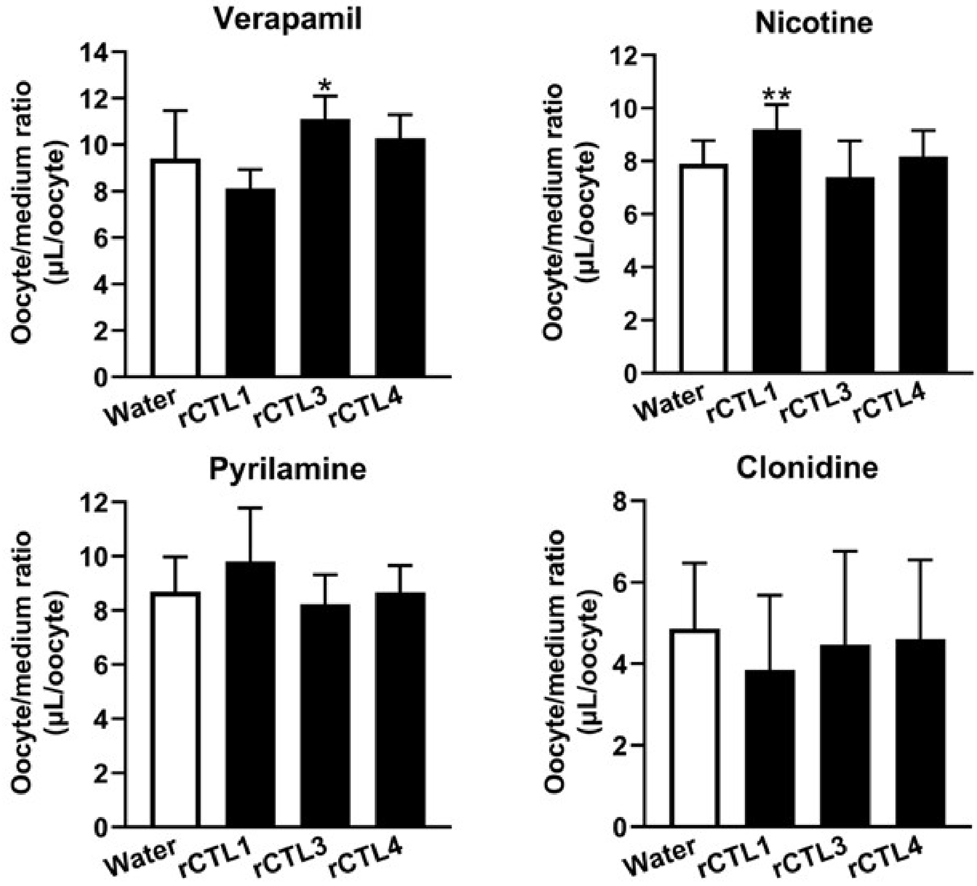

Figure S1. Uptake of cationic compounds by *Xenopus laevis* oocytes. Uptake of [^{3}H]verapamil (0.45 µCi), [^{3}H]nicotine (0.45 µCi), [^{3}H]pyrilamine (0.45 µCi), and [^{3}H]clonidine (0.45 µCi) by water-, rCTL1, rCTL3 or rCTL4 cRNA-injected oocytes was examined at 20°C for 60 min. Each column represents the mean ± S.D. (n = 9–30). *p<0.05, ** p<0.01, significantly different from water-injected oocytes.

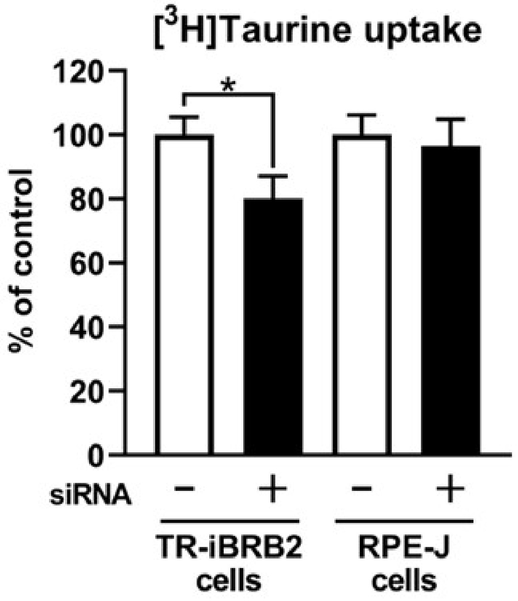

Figure S2. Effect of rCTL1 knockdown on the uptake of [^{3}H]taurine. TR-iBRB2 cells and RPE-J cells were transfected with negative control siRNA (−) or rCTL1 siRNA (+). The uptake of [^{3}H]taurine (0.1 μCi) by siRNA-transfected TR-iBRB2 cells and RPE-J cells was measured at 37°C for 10 min. Each column represents the mean ± S.D. (n = 3). *p<0.05, significantly different from negative control siRNA-transfected cells.

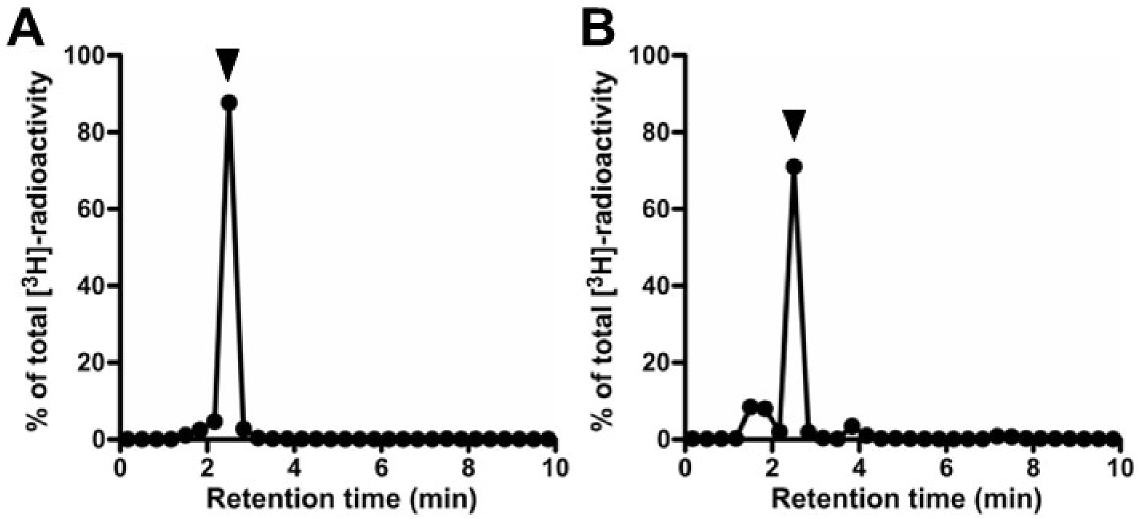

Figure S3. HPLC chromatograms of the [^{3}H]putrescine solution (A) and tissue (retina and vitreous humor) samples (B) after the microdialysis study. Retina and vitreous humor were obtained after microdialysis study and HPLC analysis was performed at a flow rate of 1.0 mL/min with Inertsil ODS-3® (3 μm) (GL Sciences Inc., Tokyo, Japan) and EP-300 (Eicom, Kyoto, Japan). Mobile phase contains 121 mM sodium acetate, 7.8 mM sodium octanesulfonate, and 22% acetonitrile. The [^{3}H]-derived radioactivity in each eluent was determined using AccuFLEX LSC7400 (Nippon RayTech Co., Ltd., Tokyo, Japan). Arrowheads indicate the retention time of [^{3}H]putrescine.

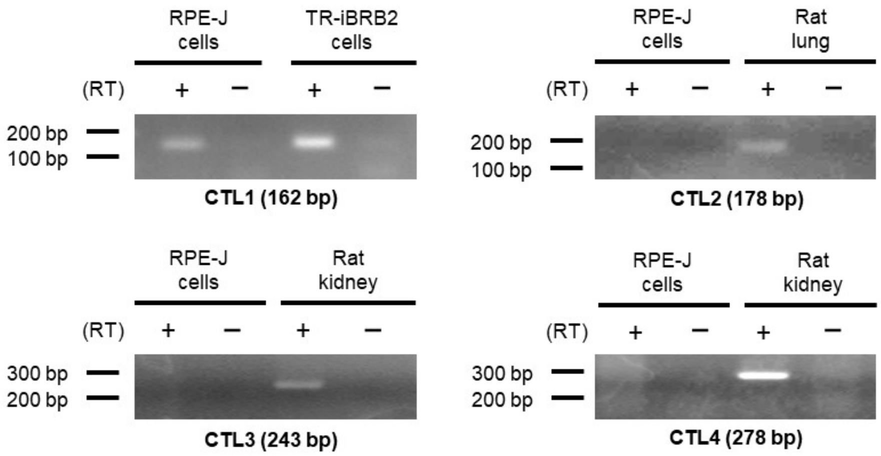

Figure S4. mRNA expression of rat CTLs in RPE-J cells. RT-PCR analysis was performed with total RNA prepared from RPE-J cells in the presence (+) or absence (−) of reverse transcriptase (RT). Total RNA prepared fromTR-iBRB2 cells, rat lung, and rat kidney was used as positive control sample. Arrowhead indicates predicted product size. Primers are shown in Table S2.
